# Local Diagnostic Reference Levels for Intracranial Aneurysm Coil-Only Embolization Using a Low-Dose Technique

**DOI:** 10.3390/biomedicines14010233

**Published:** 2026-01-21

**Authors:** Mariusz Sowa, Joanna Sowa, Kamil Węglarz, Maciej Budzanowski

**Affiliations:** 1 Department of Neurosurgery, Collegium Medicum University of Warmia and Mazury Aleja Warszawska 30, 10-082 Olsztyn, Poland; neurochirurgianeurochirurgia@uwm.edu.pl (M.S.); lekkamiladamweglarz@gmail.com (K.W.); 2 Independent Researcher, Aleja Warszawska 30, 10-082 Olsztyn, Poland; 3 Department of Radiation Physics and Dosimetry, Polish Academy of Sciences, 31-342 Kraków, Poland; maciej.budzanowski@wp.pl

**Keywords:** DRL, diagnostic reference levels, aneurysm treatment, coil-only embolization, radiation dose optimization

## Abstract

**Background/Objectives:** Optimizing routine neurointerventional workflow and minimizing exposure to ionizing radiation during coil-only endovascular treatment of intracranial aneurysms depend on operator experience, reduced frame rates during both fluoroscopy and digital subtraction angiography (DSA), and the use of advanced angiographic systems. The low-dose protocol implemented in this study used the lowest available fluoroscopy frame rate (3.125 frames per second [fps]) and a nominal acquisition rate of 2 fps (actual = 2.45 fps) for DSA, three-dimensional (3D) rotational angiography, two-dimensional (2D)/3D mapping, and roadmapping. **Methods:** This retrospective analysis encompassed 245 coil-only procedures performed at a single tertiary center from 2018 to 2024. Data collected for each procedure included dose-area product (DAP), reference air kerma (K_a,r_), fluoroscopy time (FT), and the total number of DSA frames. Local diagnostic reference levels (DRLs; 75th percentile [P75]) and typical values (50th percentile [P50]) were determined and descriptively compared with values reported in the literature. **Results:** The P75 values, representing DRLs, were 22.4 Gy·cm^2^ for DAP (literature range, 123–272.8 Gy·cm^2^), 268 mGy for K_a,r_ (1171–4240 mGy), 18 min 56 s for FT, and 285 DSA frames. The P50 values were 13.8 Gy·cm^2^ for DAP (78.7–179.0 Gy·cm^2^), 196 mGy for K_a,r_ (801–2804 mGy), 13 min 25 s for FT, and 208 DSA frames. **Conclusions:** In this single-center cohort, dose metrics for coil-only intracranial aneurysm treatment were within the lower range of published values. Cross-study comparisons are descriptive and require cautious interpretation. The proposed local DRLs may support quality assurance, dose optimization, and patient safety in comparable clinical settings. Further multi-center and multi-operator studies are warranted to evaluate transferability and applicability beyond coil-only procedures.

## 1. Introduction

Cumulative exposure to ionising radiation over a patient’s lifetime—stemming, for example, from diagnostic X-ray imaging—is associated with an increased stochastic risk, including malignancy [1,2]. Neuroendovascular therapy is inherently X-ray guided; therefore, radiation output during intracranial interventions should be minimised to the lowest level compatible with procedural safety and adequate imaging performance [3,4] and the availability of contemporary angiographic technology [5], and the availability of contemporary angiographic technology [6]. At our institution, these optimisation measures include the use of very low fluoroscopy frame rates (3.125 frames per second) and low default acquisition settings (2 frames per second) for DSA and ancillary modes (e.g., 3D rotational angiography, 2D/3D mapping and roadmapping), supported by vendor-specific processing (ClarityIQ, Philips Healthcare, Cambridge, MA, USA) to maintain clinically acceptable image quality at reduced exposure [7]. In addition, the operator uses the ClarityIQ system (Philips Healthcare) to reduce dose and enhance image quality.

Radiation indicators recorded during neurointerventional procedures are shaped by a combination of patient-related and workflow-related factors. To support systematic optimisation and benchmarking, diagnostic reference levels (DRLs) are commonly used as pragmatic dose-management tools, providing distribution-based reference values for defined imaging tasks. The present study derives local typical values (median; P50) and local DRLs (P75) for X-ray-guided cerebral endovascular procedures performed for intracranial aneurysms using coil embolisation alone. We included only cases without adjunctive devices (e.g., stents or flow diverters). A single-centre retrospective analysis was conducted for procedures performed between 2018 and 2024; patient sex, body weight and age were recorded, and our results were contextualised against reports published between 2013 and 2025.

## 2. Materials and Methods

The study used retrospective data collected between 2018 and 2024 and, in accordance with applicable regulations, did not require approval by the Bioethics Committee. A total of 245 patients (170 women and 75 men) who underwent endovascular treatment of intracranial aneurysms using coil embolization alone at the University Hospital in Olsztyn were included in the analysis. Eligible patients were aged 26 to 92 years, with a mean age of 59 (Table 1).

Procedures were categorized into two main groups: unruptured intracranial aneurysms (UIA, *n* = 130) and ruptured intracranial aneurysms (RIA, *n* = 115). Further subgroups were defined based on patient characteristics (sex) and aneurysm location (Supplement Table S1). Cases were also classified according to the number of X-ray tubes/projections used during treatment (single/frontal only vs. dual/frontal + lateral) (Supplement Table S2).

In accordance with the recommendations of the International Committee on Radiological Protection (ICRP) Publication 135 [8], all available data were included in the analysis. Owing to the large variability and high standard deviation (SD), a logarithmic transformation of the data was performed, which resulted in an approximately normal distribution.

### 2.1. Statistical Analysis

Statistical analysis was conducted in Python 3.12 using the pandas 2.3.3 and SciPy libraries 1.16.2, and visualizations were generated with matplotlib 3.10.3. Due to the observational and retrospective nature of the study, which focused on dose auditing and the establishment of local DRLs, the analysis relied primarily on descriptive methods and on the assessment of dose metric distributions.

Continuous variables were reported as the median (P50) and interquartile range (IQR, P25–P75), along with selected percentiles; P75 was defined as the local DRL. Normality was assessed using the Shapiro–Wilk test. Homogeneity of variances was evaluated for parametric analyses; when this assumption was violated, Welch’s *t*-test was applied.

Comparisons between two independent groups were performed using the Mann–Whitney U test when normality assumptions were not met. For data approximating a normal distribution, Student’s *t*-test was used, or Welch’s *t*-test when variances were unequal. All tests were two-sided, and *p* ≤ 0.05 was considered statistically significant. Subgroup analyses were treated as exploratory and descriptive.

### 2.2. Low-Dose Technique

All endovascular procedures employed a strategy that prioritized maximal frame rate reduction in both fluoroscopy and DSA modes, in conjunction with modern angiographic equipment. Extensive experience with reduced acquisition parameters was also essential. The operator acquired this expertise through prolonged observation and training under a specialist who was accustomed to working with low frame rates [9]. During the initial five years of a twelve-year clinical practice, the operator used equipment with lower performance than the current Azurion system, which has been in operation since 2018. This experience increased the operator’s tolerance for less smooth image sequences associated with low frame rates (reduced temporal resolution), rather than poorer image quality, and enhanced the spatial perception required for precise visualization of vascular pathology. As described in Section 2.3, regular quality control (QC) testing confirms that image quality at these low-dose settings meets diagnostic requirements for neuroangiographic imaging.

**Figure 1:**
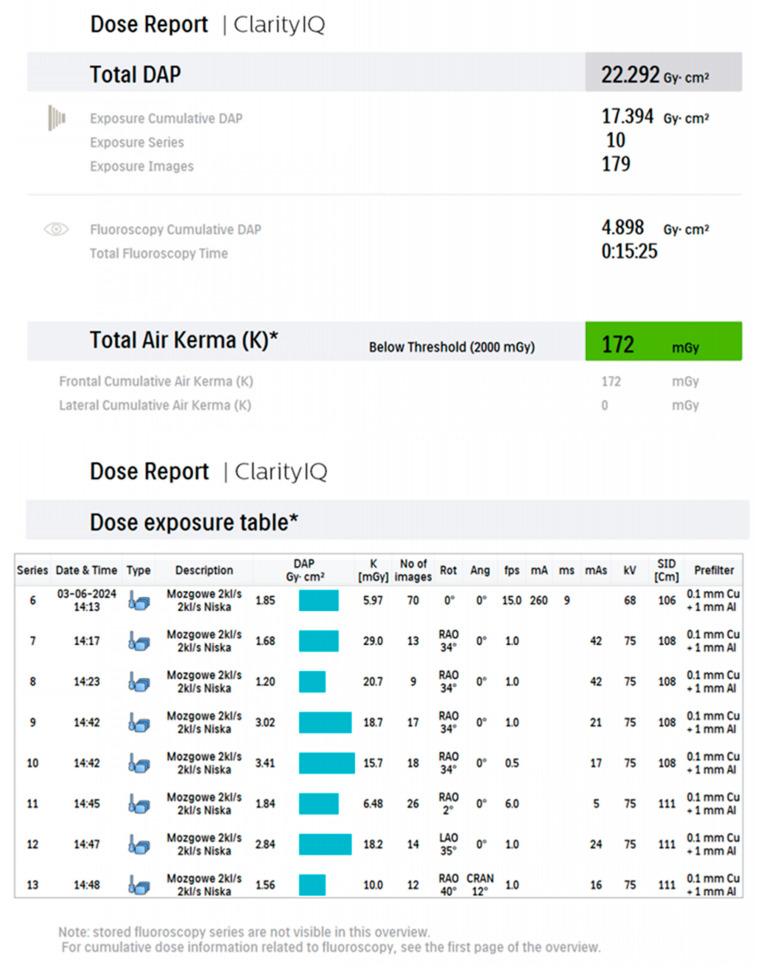
Example dose report from coil-only treatment of an intracranial aneurysm using a single-plane system (patient X) * Air kerma is reported at the interventional reference point (IRP), 15 cm from the isocenter towards the tube.

Nominal frame rates of 2 fps for DSA and 3.125 fps for fluoroscopy were established as baseline system settings, enabling the lowest feasible radiation dose during the procedure (Figure 1). Additionally, the nominal DSA frame rates were modified by automatic exposure adaptation (LOW mode), which was activated by the operator. The “Description” column of the acquisition report, containing the entry in Polish “mózgowe 2 kL/s, 2 kL/s niska” (“Cerebral 2 fps, 2 fps Low”), confirms the selection of LOW mode. In this mode, Azurion algorithms, which consider patient tissue thickness, geometric configuration (projection angles), filtration, and other input parameters, automatically adjust the frame rate and exposure parameters to maintain sufficient image quality while minimizing radiation dose. This approach reduces radiation exposure for both patients and medical staff.

The dynamic behavior of the dose-optimization algorithm in LOW mode is illustrated in Figure 1. For example, in Figure 1, DSA series 6 started at 15 fps, after which the system reduced the frame rate to approximately 1 fps. In series 9 and 10, despite identical nominal settings and projection angles, the frame rate dropped to ~0.5 fps. After changing the tube angle in series 11, the frame rate increased to around 6 fps. In series 12–13, it returned to approximately 1 fps. These fluctuations reflect algorithm-driven adaptation to changing imaging conditions.

To characterize the exposure settings achieved in clinical practice, we retrospectively extracted technical parameters from the system dose reports for all procedures included in the study. For the DSA series, we calculated the mean realized tube voltage (kV), tube current–time product per frame (mAs), filtration (Sr), and effective frame rate. The corresponding values for the whole cohort were

− mAs: 30.86 mAs ± 14.42/1–64
− kV: 74.23 kV ± 2.83/61–80
− Sr: 0.1 mm Cu + 1 mm Al, for almost all cases fps: 2.45 ± 4.08/0.5–15.

For fluoroscopy, in addition to the total fluoroscopy time (FT), we used the dose report to extract the associated dose-area product (DAP) values for all procedures:

− FT in seconds: 935.6 ± 478.1/310–4324
− fluoroscopy-related DAP: 6.048 ± 2.8 84/1.47–16.541

These data demonstrate that the low-dose strategy was reflected in the nominal settings and the actual exposure parameters used in routine clinical practice.

Radiation exposure during a given procedure also depended on the number of X-ray tubes (projections) required to visualize the vascular lesion. Whenever feasible, procedures were performed in single-plane projection only, whereas biplane imaging was reserved for more complex cases (e.g., unfavorable aneurysm location, vessel tortuosity, or complex aneurysm morphology). The impact of single-plane versus biplane imaging on DAP, K_a,r_, and frame counts is summarized in Supplement Table S2.

### 2.3. Angiographic System and Quality Control

A structured quality QC program was implemented in our angiography suite in accordance with the manufacturer’s recommendations and national QC guidelines. While the monthly QC routine does not include formal signal-to-noise-ratio (SNR) measurements, the angiography system automatically stores technical image data during clinical procedures.

As part of a retrospective analysis, SNR was calculated for DSA images acquired with the standard low-dose protocol in 15 consecutive patients undergoing treatment for intracranial aneurysms. For each case, a circular region of interest (ROI) was placed within a high-contrast intracranial internal carotid artery (ICA) segment (signal), and a second ROI was positioned in a homogeneous background area (noise). SNR was defined as the mean pixel value in the signal ROI divided by the SD of pixel values in the background ROI:

$$SNR\;=\frac{Mean\;signal}{SD\;background}$$

Across the analyzed cases (*n* = 15), the mean DSA SNR was 52.7 (SD 23.0). This quantitative assessment, provided as Supporting information, indicates that image quality remained adequate at low-dose settings. However, SNR alone does not constitute comprehensive validation of diagnostic performance or clinical outcomes, and the present study is primarily focused on dose distributions and the establishment of local DRLs.

### 2.4. Three-Dimensional Rotational Angiography

Three-dimensional rotational angiography (3D-RA) on the Azurion platform is performed using a partially automated workflow. After the operator initiates the run, the C-arm executes a predefined rotational trajectory while images are acquired automatically, followed by on-system volumetric reconstruction. Before acquisition, the operator selects the programme and sets the key parameters (e.g., dose level, frame rate, and rotation range) together with the contrast injection settings.

In our centre, 3D-RA is obtained using a standardised Azurion protocol with fixed geometry and acquisition settings (122 images, LAO 103°, 30 fps, SID 120 cm). Despite identical nominal parameters, the recorded dose indicators (DAP and K_a,r_) may differ slightly between runs, reflecting inter-patient variability (e.g., habitus, positioning and attenuation) and the action of the automatic exposure control, which modulates tube output (including tube current and pulse characteristics) to preserve image quality at the lowest achievable exposure. Of the 245 procedures, 79 cases (32.2%) used 3D-RA. 3D-RA was performed once per procedure in 46 cases, twice per procedure in 29 cases, and four times per procedure in two cases.

For each 3D-RA acquisition, the mean DAP and reference point air kerma (K_a,r_) were 3.99 ± 0.70 Gy·cm^2^ and 11.1 ± 2.2 mGy, respectively. Most of the 3D-RA acquisitions—92.4% (73 out of 79)—were performed using single-plane imaging (Supplement Table S2).

### 2.5. Computed Tomography

By default, a control computed tomography (CT) scan was performed 24 h after the therapeutic procedure. A routine non-contrast head CT was performed within 24 h after endovascular treatment to allow early detection of potential post-procedural complications. The primary aim of this control imaging was to exclude acute intracranial hemorrhage, cerebral infarction, or significant brain edema that could result from the intervention. Additionally, the follow-up CT confirmed the stability of the embolic material and provided a baseline for subsequent neurological changes. The angiography suite, which houses the angiography system, is a hybrid operating room equipped for both endovascular and neurosurgical interventions. In selected cases, a CT scan was obtained intra-procedurally. In 15 of 245 procedures, CT was performed during the intervention: in eight cases due to ventricular drainage implantation, due to suspected intraoperative bleeding in five, and suspected vasospasm in two.

The mean DAP per CT acquisition was 28.4 ± 0.4 Gy·cm^2^, and K_a,r_ was 67.5 ± 1.3 mGy.

**Figure 2:**
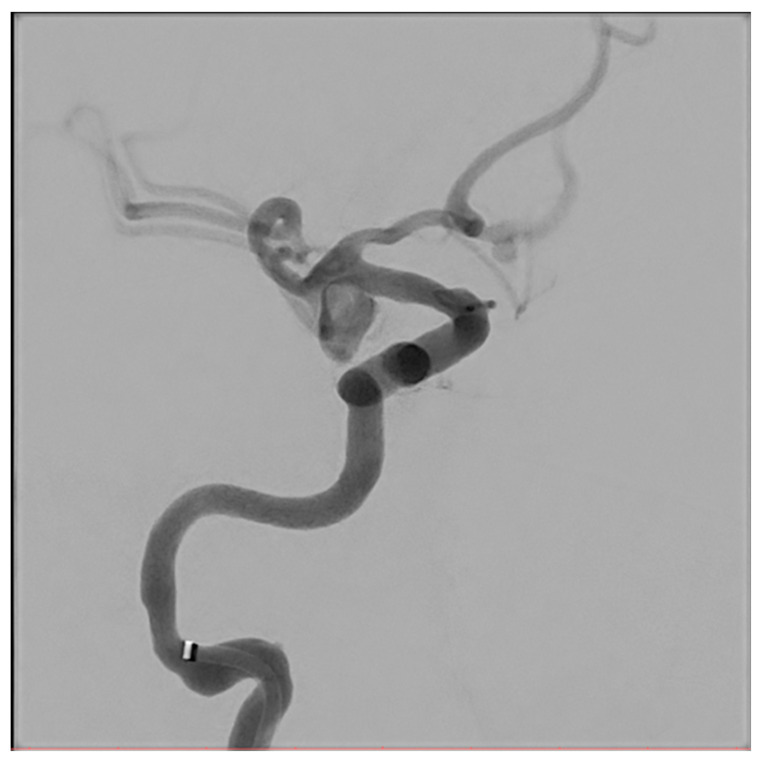

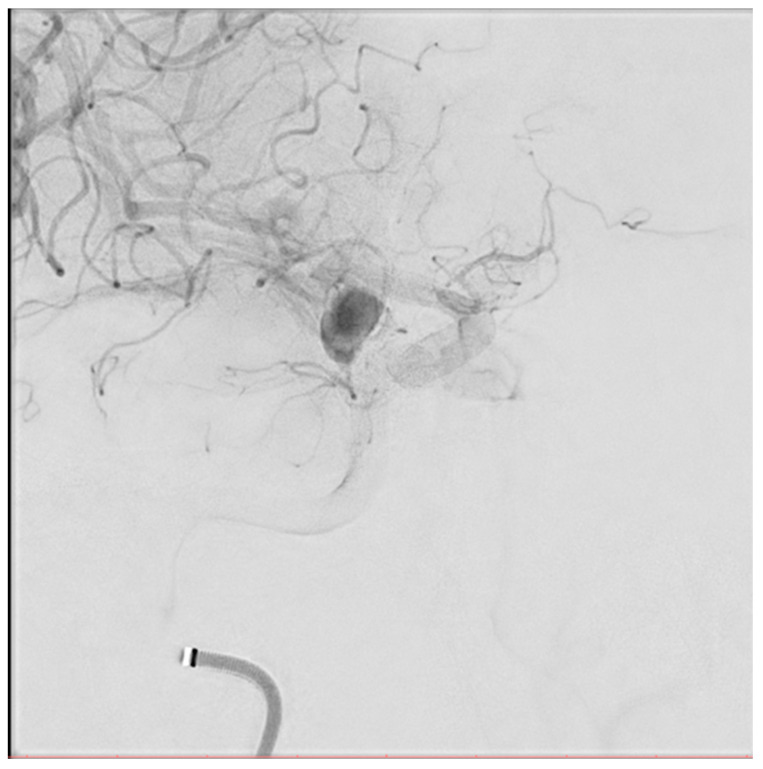
Angiography of an internal carotid artery aneurysm (patient X).

**Figure 3:**
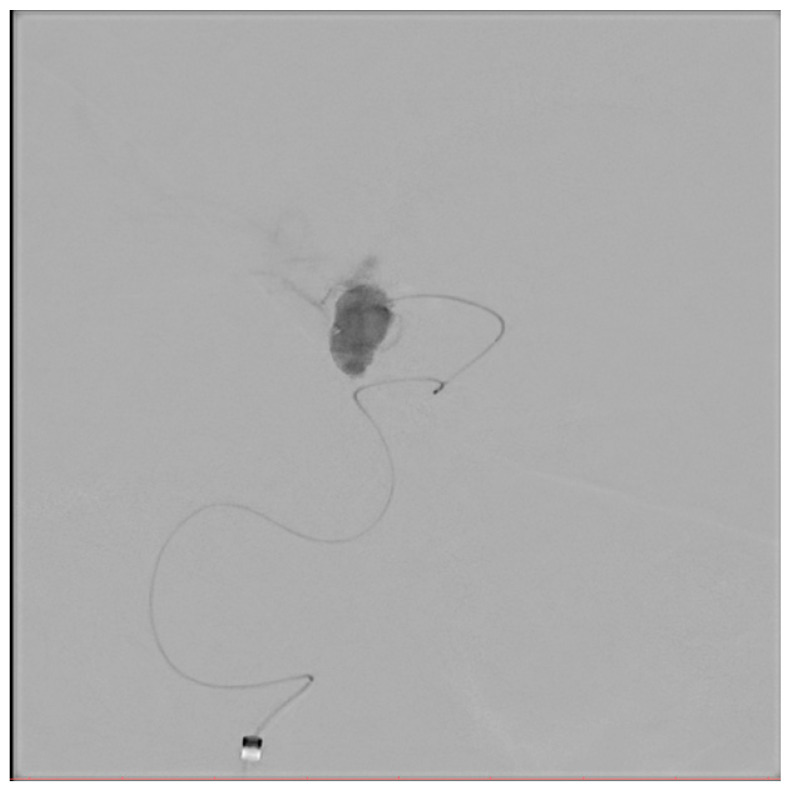
Aneurysmography of an internal carotid artery aneurysm (patient X).

**Figure 4:**
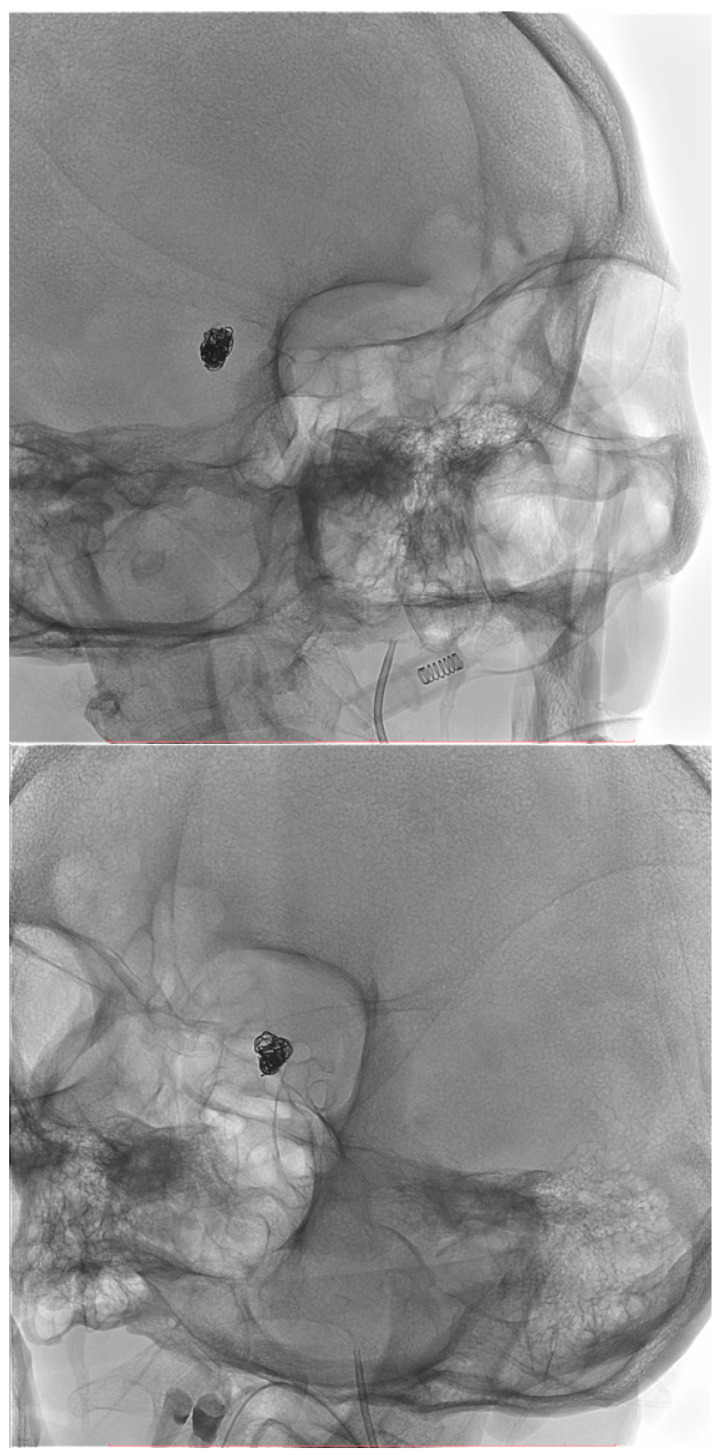

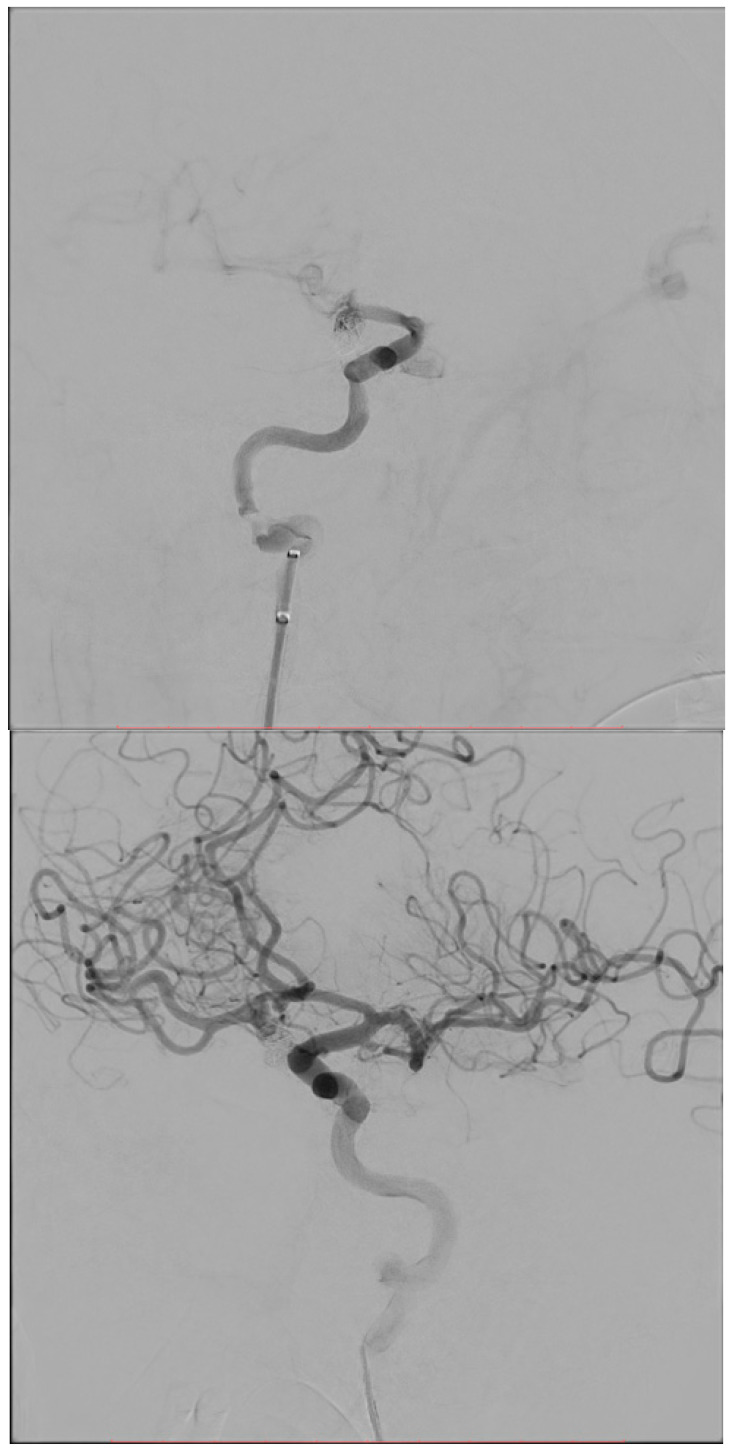
Internal carotid artery aneurysm completely occluded with coils (patient X).

**Figure 5:**
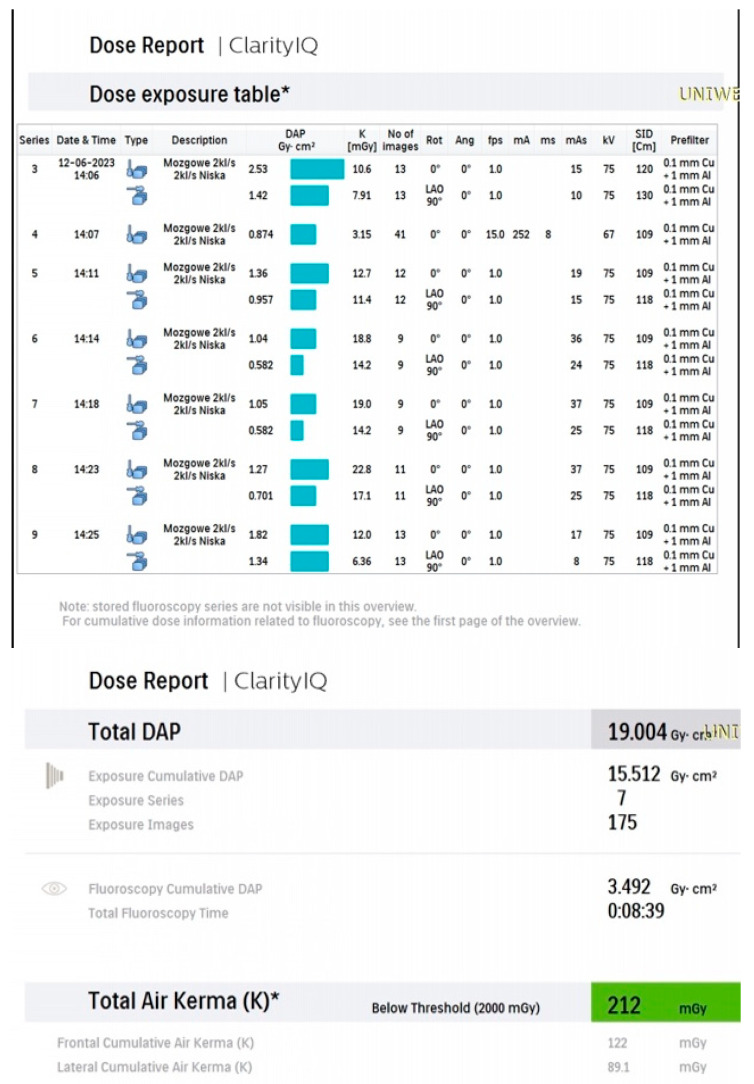
Example dose report from coil-only treatment of an intracranial aneurysm using a b-plane system (patient Y) * Air kerma is reported at the interventional reference point (IRP), 15 cm from the isocenter towards the tube.

**Figure 6:**
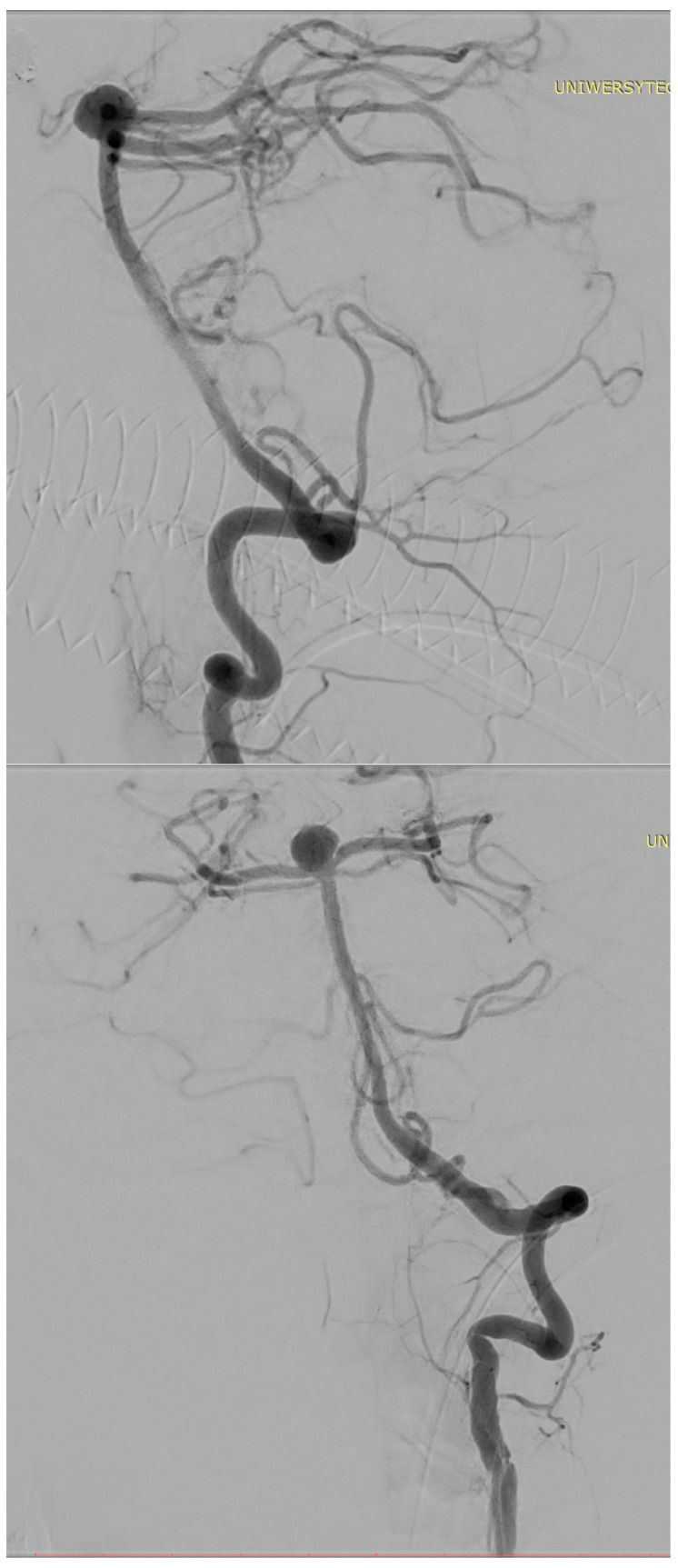
Angiography of a basilar artery aneurysm (patient Y).

**Figure 7:**
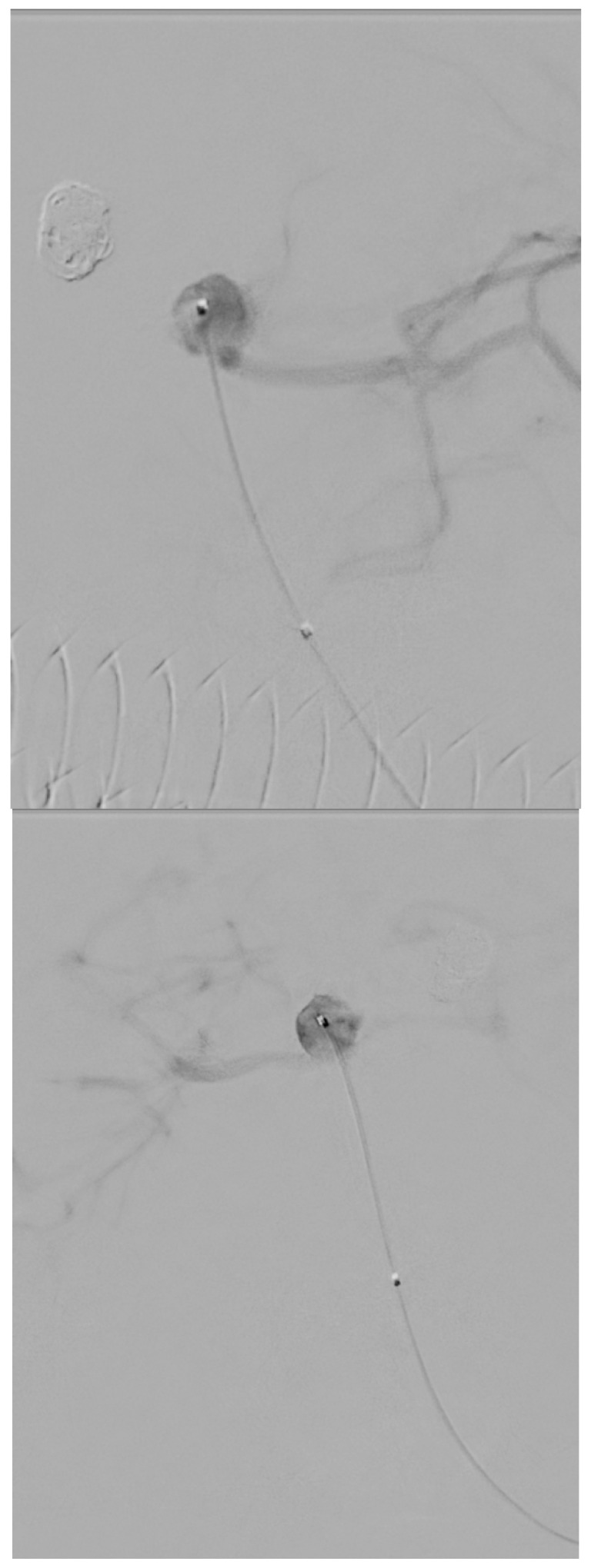
Aneurysmography of a basilar artery aneurysm (patient Y).

**Figure 8:**
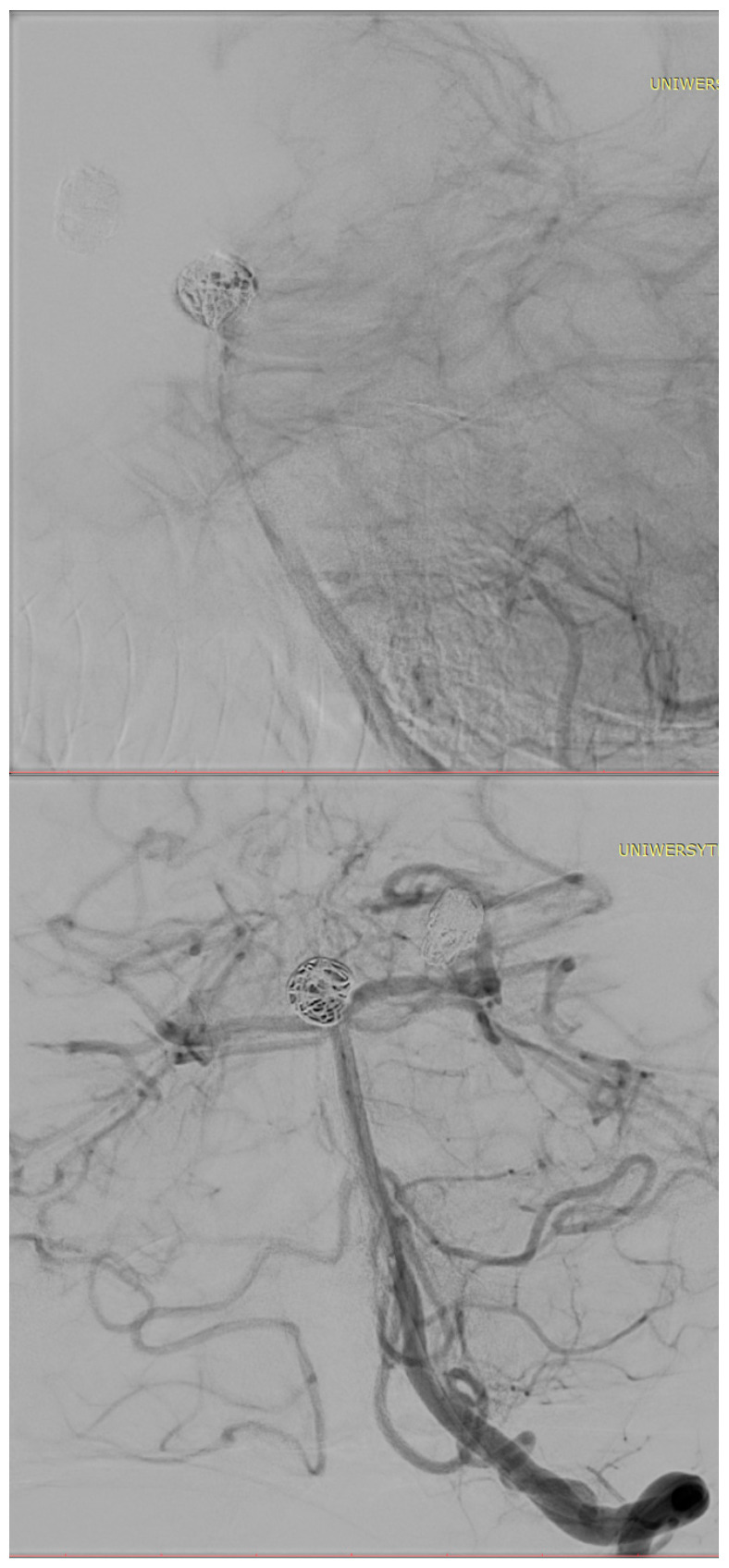
Frontal and lateral views on follow-up angiography after coil-only treatment of a basilar artery aneurysm (patient Y).

Figure 1, Figure 2, Figure 3, Figure 4, Figure 5, Figure 6, Figure 7 and Figure 8 present representative data from two patients (patient X and patient Y) who underwent endovascular intracranial aneurysm treatment using a coil-only technique (embolisation with coils alone). For each case, the dose report and selected procedural images are shown. In patient X (Figure 1, Figure 2, Figure 3 and Figure 4), the intervention was performed using a single X-ray tube (single-plane), whereas in patient Y (Figure 5, Figure 6, Figure 7 and Figure 8) a biplane configuration with two X-ray tubes was used, enabling simultaneous acquisition in two orthogonal projections.

## 3. Results

Table 2 summarizes descriptive statistics for the entire cohort of 245 intracranial aneurysm coil embolization procedures. It reports typical values (median, P50), local DRL’s, and the mean ± SD for DAP, K_a,r_, FT, and the number of DSA frames per procedure. Results are presented for the overall cohort and stratified by sex (female vs. male), aneurysm status (ruptured [RIA] vs. unruptured [UIA]), and the number of angiographic projections (frontal-only vs. combined frontal and lateral). In addition, the table includes between-subgroup comparisons, reported as *p*-values for statistical differences across the respective subgroups. More detailed subgroup analyses by sex and projection type within the RIA and UIA groups, including additional descriptive statistics, are provided in Tables S3 and S4. Overall, the DRL (P75) for coil-only endovascular treatment of intracranial aneurysms was 22.4 Gy·cm^2^ for DAP, 268.0 mGy for K_a,r_, 18 min 58 s for FT, and 285 for the number of DSA frames.

Typical values (P50) were 13.8 Gy·cm^2^ for DAP (literature range, 78.7–164.5 Gy·cm^2^), 196 mGy for K_a,r_ (literature range, 801–2197 mGy), 13 min 28 s for FT (literature range, 19.5–51.4 min), and 208 for the number of DSA frames.

As shown in Supplemental Tables S2 and S4, DAP (*p* < 0.05) and K_a,r_ (*p* < 0.05) were significantly higher in men than in women, whereas FT and the number of DSA frames did not differ significantly between these subgroups.

In both women and men, K_a,r_ values were significantly higher in RIAs than in UIAs, while no statistically significant differences in DAP were observed between these groups.

In women, both FT and the number of DSA frames per procedure were significantly lower in RIA cases than in UIA cases. In men with ruptured aneurysms, only FT was significantly lower compared to men with UIAs, whereas the number of DSA frames did not differ significantly.

These findings support the hypothesis that emergency procedures performed for aneurysmal rupture are likely shorter than elective procedures; however, this hypothesis requires confirmation in further studies.

The biplane system (frontal and lateral C-arms) was used in 150 of 245 intracranial aneurysm embolization procedures (61.2%). The mean DAP distribution between the frontal and lateral C-arms was 67% and 33%, respectively (Supplement Table S2).

Radiation exposure in procedures performed in frontal projection only was significantly lower than in procedures in which both frontal and lateral projections were used. As shown in Supplement Table S2, the vast majority of 3D-RA acquisitions (92.4%; 73/79; *p* < 0.05) were performed using single-plane imaging. In these cases, the operator relied on 3D maps and roadmapping for spatial visualization of the vascular lesion, thereby obviating the need for an additional lateral projection. On this basis, it can be hypothesized that the use of 3D-RA may reduce radiation exposure by decreasing the number of projections required.

Similar findings and conclusions regarding the use of 3D-RA were reported by the present author in a study on local DRLs for diagnostic cerebral angiography [10], in which 3D-RA, when used instead of conventional angiography to visualize more than one vessel, resulted in a significant reduction in patient radiation exposure.

These results are consistent with the notion that radiation usage depends on the number of projections required for reliable visualization of the vascular pathology. The decision to use single-plane versus biplane imaging was also driven by clinical and anatomical considerations (spatial location, vessel diameter and course, and aneurysm morphology), which, in turn, explain the higher doses in more complex cases. The collected data suggest that greater aneurysm morphological complexity may be associated with higher patient exposure to X-rays; this, however, also requires confirmation in future studies.

Statistical comparisons within the UIA and RIA subgroups for procedures performed in frontal projection only were not carried out because the number of RIA cases (*n* = 22) was too small for a reliable analysis. Further comparative analyses will be performed once a statistically adequate sample has been obtained.

Supplement Table S5 presents the results for aneurysm treatment stratified by location in the anterior versus posterior circulation of the circle of Willis and by rupture status (UIA vs. RIA). Supplement Table S6 summarizes the probability analyses for differences between these subgroups (anterior vs. posterior circulation and UIA vs. RIA) with respect to DAP, K_a,r_, FT, and the total number of DSA frames. Based on the data presented in Supplement Tables S5 and S6, no statistically significant differences in DAP, K_a,r_, or FT were found between aneurysms located in the anterior and posterior circulations of the circle of Willis.

By contrast, there were statistically significant differences between ruptured and unruptured aneurysms, regardless of their location. These differences were also evident within the UIA (unruptured intracranial aneurysms) and RIA (ruptured intracranial aneurysms) subgroups, both in the anterior and posterior parts of the circle of Willis (Supplement Tables S5 and S6).

For ruptured aneurysms located in the anterior circulation, significantly higher Ka, r values (251.9 ± 135.8 vs. 199.0 ± 118.5 mGy and 271.7 ± 158.3 vs. 192.7 ± 131.7 mGy) and a higher number of frames per procedure were observed, together with significantly shorter FT (851.4 ± 447.1 vs. 1028.7 ± 526.2 s and 749 ± 283.6 vs. 1077.3 ± 480.1 s), compared with unruptured aneurysms.

The results in Tables S5 and S6, therefore, confirm that stratification by rupture status reveals statistically significant differences between ruptured and unruptured aneurysms. It should be noted, however, that the UIA subgroup in the posterior circulation comprises only 28 cases, which may limit the robustness of the statistical analysis. Larger patient cohorts are therefore needed to validate these findings.

The observed differences may be related to the fact that treatment of ruptured aneurysms is performed as a life-saving emergency procedure and is intended to be completed as quickly as possible, which can influence both exposure parameters and procedural workflow. Nonetheless, this issue warrants further investigation and confirmation in a larger clinical series.

## 4. Discussion

Advances in modern technology and the widespread availability of imaging studies have made ionizing radiation-based methods some of the most commonly used tools in routine diagnostic and therapeutic practice [11,12]. However, this is associated with an increased risk of stochastic effects, such as cancer [13,14], and deterministic effects, including cataracts and radiation-induced skin injuries [15,16], which represent an unavoidable “trade-off” of life-saving interventional procedures.

Consequently, the establishment of local DRLs for centers that routinely perform endovascular procedures has become standard practice in recent years. Based on the Euratom Basic Safety Standards Directive and ICRP Publication 135, the DRL concept is now well established and clearly defines the principles for determining these levels [8]. This enables operators to assess whether the radiation dose falls within the typical range of dose distributions or whether further exposure optimization is required.

In the present study, we proposed local DRLs for coil-only endovascular treatment of intracranial aneurysms. This work was designed as a retrospective dose audit and DRL analysis based on routinely reported dose metrics (DAP, K_a,r_, FT, and number of DSA frames), rather than a clinical outcomes study or a controlled protocol-comparison study. When viewed in the context of previously published data summarized in Table 3, the DRL values obtained in our cohort fall within the lowest range of reported dose metrics. These differences, however, should be interpreted with caution. Our intention was not to perform formal statistical hypothesis testing between our cohort and external datasets, as direct statistical comparisons of independent studies—conducted in different centers, with different inclusion criteria, case mix, operators, and equipment—are not appropriate. The comparisons presented here are therefore descriptive only and intended to position our results within the range of values reported in the literature rather than to claim statistically proven superiority.

Achieving relatively low exposure levels in the analyzed cohort is likely due to a combination of factors, including the operator’s extensive experience with low-frame-rate imaging systems and the use of advanced dose-reduction technologies. The applied technique is described in detail in Section 2. All procedures were performed in a single high-volume neurointerventional centre by one experienced neurointerventionalist with approximately 12 years of clinical practice at the time of the study, using a modern biplane angiography system equipped with dedicated low-dose options. This single-centre, single-operator setting reduces intra-study variability but, at the same time, limits the generalizability of our findings to centers with different case-mixes and operator experience. Accordingly, the reported DRLs should be interpreted as local reference values that may reflect a highly specialized (“best-case”) practice environment, rather than a population-level benchmark. In addition, all procedures were performed on a single biplane angiography system with vendor-specific dose-reduction and dose-optimization features, which may limit generalizability across different platforms and configurations.

Proficiency in working at reduced frame rates should be developed under the supervision of an experienced operator who has already mastered this approach. Observing procedures performed at reduced frame rates provides an excellent basis for the subsequent independent use of this technique. The learning curve for this approach is not quantified in the present retrospective dataset. It may vary between operators, requiring both substantial hands-on experience and an in-depth knowledge of neuroanatomy, including the cerebral vasculature. For this reason, it is not recommended that novice operators markedly reduce frame rates early in their careers. Therefore, implementation should be accompanied by structured training and routine QC to ensure stable system performance and consistent workflow.

The present study focused on assessing DRLs for coil-only endovascular treatment of intracranial aneurysms. In addition, the analysis was stratified by the patient’s baseline clinical status: elective treatment of UIAs versus emergency treatment of RIAs. These subgroup comparisons are presented as descriptive/exploratory and should not be interpreted as causal effects, as key determinants of dose (e.g., aneurysm morphology and procedural complexity) were not available in a structured manner for multivariable adjustment. To the best of our knowledge, a similar stratification into UIA and RIA has been applied by Opitz et al. [17] (Table 3) only so far. In their study, the DRL, defined as P75 of DAP, was 217 Gy·cm^2^ for the entire cohort of coil-only aneurysm treatments, 183 Gy·cm^2^ for UIAs, and 246 Gy·cm^2^ for RIAs. It should be noted that the time span of procedures included in that analysis was broad; data collected between 2010 and 2021 may differ substantially in terms of exposure levels due to the rapid evolution of endovascular techniques and equipment over the past decade.

Forbrig et al. [18] reported data on patients with saccular UIAs and analyzed various endovascular treatment techniques. The study included only selected aneurysm locations: the anterior communicating artery (ACom), intradural segments of the ICA, including the posterior communicating artery, and the apex of the basilar artery (BA). The authors identified a subgroup of UIAs treated with coils alone (*n* = 23) and estimated the DRL at 130 Gy·cm^2^.

In a multicenter study, Ihn et al. [19] reported data on radiation exposure during diagnostic and therapeutic procedures for intracranial aneurysms performed with modern biplane angiography systems. The reported DRL for coil-only endovascular treatment of intracranial aneurysms was 199.9 Gy·cm^2^, and the P75 for Ka, r was 3458.7 mGy. Both values are higher than those observed in our cohort, but it must be emphasized that the authors did not stratify the data by embolization technique or by rupture status (UIA vs. RIA). Their dataset, collected over the last five years from multiple centers and operators, reflects an average DRL performance in a heterogeneous clinical environment rather than that of a single specialized center.

Rizk et al. [20] also evaluated dosimetric parameters according to the endovascular treatment technique. They reported some of the lowest values among the studies summarized in Table 3 (P50 for DAP of 91 Gy·cm^2^ and P50 for Ka, r of 1113 mGy).

Descriptively, the DRL values for DAP obtained in the present study are several-fold lower than those reported by Opitz et al. [17], Ihn et al. [19], and Rizk et al. [20]. In our cohort, the P75 for DAP was 22.4 Gy·cm^2^ for all coil-only aneurysm treatments, 22.3 Gy·cm^2^ for UIAs, and 22.7 Gy·cm^2^ for RIAs. Although the differences in DAP between UIAs and RIAs were not statistically significant, other parameters—including Ka, r, FT, and the number of DSA frames—were significantly lower in the RIA group. These inter-study differences in absolute values must, however, be interpreted in light of the methodological heterogeneity discussed above.

The remaining publications summarized in Table 3 predominantly concern endovascular treatment of intracranial aneurysms in general, without differentiating between specific techniques. Against this background, the results obtained in our study are clearly lower. The P75 for DAP was 22.4 Gy·cm^2^, and the P75 for Ka, r was 268 mGy. By comparison, the P75 values for DAP reported in Table 3 range from 123 to 272.8 Gy·cm^2^, while the corresponding P75 values for Ka, r range from 1171 to 4240 mGy. These differences likely reflect, at least in part, variations in case-mix, operator experience, aneurysm complexity, and the use of more complex techniques such as stent-assisted coiling, flow diverters, or balloon-assisted techniques.

Such large differences may be related, among other factors, to the FT required to visualize the vascular lesion. In our study, the P75 for FT was just under 19 min, whereas in the studies included in Table 3, the corresponding values ranged from 35 to 64.7 min. This may reflect the fact that coil-only treatment is a fundamental technique that is less time-consuming than procedures involving coils and stents, flow diverters, or balloon-assisted techniques, which require longer procedure times.

As shown by other authors [7,20,21], radiation dose in interventional X-ray procedures is influenced by multiple factors, including procedural complexity, the specific endovascular technique used, the experience of the medical staff, and the angiographic system settings. For this reason, the present study focused on aneurysms treated exclusively with coils to improve the dosimetric homogeneity of the collected data.

Analysis of Table 3 shows that the available data on X-ray exposure during endovascular treatment of intracranial aneurysms span the years 2010–2021. This may partly explain the higher exposure values, given the continuous development of micro-instrumentation, endovascular techniques, and angiographic equipment. In the present study, procedures performed between 2018 and 2024 were analyzed, which most likely also contributed to the lower dose levels observed in light of these factors.

When aneurysm location is stratified into the anterior and posterior circulations of the circle of Willis, our findings are consistent with previous reports that did not demonstrate significant differences in DAP or FT between these locations [19,21]. However, these observations need to be confirmed in studies including larger patient cohorts, preferably in a multicenter setting.

Rizk et al. [20] also stratified patients by sex; however, this analysis included all types of aneurysm embolization techniques. In that study, the difference between women and men was also statistically significant (*p* = 0.03), with men exhibiting significantly higher DAP and Ka, r values than women. In our study, sex- and location-based subgroup comparisons are interpreted cautiously as descriptive findings that may be confounded by unmeasured anatomical and procedural complexity factors.

As early as 2013, Söderman M. et al. [22,23] demonstrated that modern X-ray imaging technology, combining image noise-reduction algorithms with optimized system settings, can achieve an approximately 60% dose reduction in neuroangiography and interventional neuroradiology without affecting physicians’ working habits. The results reported by Söderman and colleagues reflect the dose-reduction potential also demonstrated in the present study.

Multiple determinants contribute to the radiation output delivered during a neuroendovascular session, including patient-related factors (e.g., body habitus and vascular anatomy), clinical context, technical settings, and procedural efficiency [7]. From a technical perspective, dose is influenced by the number of angiographic acquisitions, acquisition and fluoroscopy frame rates, collimation strategy, detector technology, and geometric conditions—particularly the source-to-image distance (SID) [7]. Operator practice is also relevant; unnecessary fluoroscopy during phases without wire/catheter movement can be avoided, and targeted optimisation strategies may be introduced [24]. For effective optimisation, operators should be familiar with system specifications and default acquisition settings [25]. Notably, dose savings have been reported after adjusting manufacturer “factory” configurations of angiographic systems [26].

Published data also indicate that lowering fluoroscopy frame rates (e.g., from 15 to 7.5 frames per second) is associated with a meaningful reduction in radiation exposure for both patients and staff [7,25,27]. In addition, flat-panel detector technology may reduce exposure compared with older detector generations [28]. Further reductions may be achieved by limiting acquisitions to clinically necessary vessels and projections, guided by information from prior imaging (CTA, MRA and/or previous DSA), thereby avoiding repeated evaluation of regions such as the aortic arch and carotid bifurcations [29]. This approach supports a more focused workflow, particularly during follow-up procedures, by concentrating on vessels supplying the known lesion.

Overall, our findings suggest that substantial radiation dose reduction in coil-only intracranial aneurysm embolization is achievable on a contemporary biplane angiography platform by combining very low fluoroscopy and DSA frame rates with dedicated dose-optimization features, within our routine coil-only workflow. Although we did not perform a formal blinded multi-reader image-quality study, a retrospective SNR assessment in a small consecutive subset (*n* = 15) was provided as supportive quantitative information. It should not be interpreted as a standalone validation of diagnostic performance or clinical outcomes. Nevertheless, radiation metrics in neurointerventional procedures are strongly influenced by operator experience, equipment generation, and configuration, and case selection; therefore, cross-study comparisons should be interpreted cautiously. Future multicenter and multi-operator studies are needed to validate transferability, assess variability between operators, and evaluate applicability beyond coil-only cases.

**Table 3:** DRL studies for cerebral embolization.

Authors	Period of Collected Treatments	Number	Study	DAP (Gy·cm^2^)		K_a,r_ (mGy)		FT (min)		Nuber of Frames	
				P50	P75	P50	P75	P50	P75	P50	P75
This study	June 2018–December 2024	245	local	13.8 All 14.9 RIA 13.4 UIA	22.4 All 22.7 RIA 22.3 UIA	196.0 All 218 RIA 168.5 UIA	268.0 All 326.5 RIA 259.5 UIA	13 min, 25 s	18 min, 58 s	208	285
Hassan A.E et al. (2017) [30]	2015	71	local	78.7		1040		25.7		300	
Kanda. R. et al. (2021) [31]	-	-	nacional		210		3100				
Ihn, Y. et al. 2021 [19]	December 2020– June 2021	327	local	130.6	199.6	2104	3458.7	40.9	57.3		
Ihn Y et al. 2016 [32]	October 2016–December 2016	371	multicenrer	179.0	271.0	2804.0	4471.3	44.5	64.7	412.5	567.3
Tristram. J. et al. (2022) [33]	June 2015–April 2018	129	local	132.8	186.8	1397	1906	51.4	70		
Acton. H. et al. (2018) [34]	November 2014–September 2016	109	local	100	123						
Aly. A et al. 2024 [35]	January 2019–March 2020	local	local	85	124	801	1171	19.5	35	717	1374
Opitz. M. et al. (2023) [17]	2010–2021	583	local	157 All 125.5 UIA 187.2 RIA	217 All 183 UIA 246 RIA	-	-	32.7 all 29.2 UIA 36.3 RIA	58		
Schegerer A et al. 2019 [36]	2016–2018		local	121	192			34	54		
Forbrig et al. 2020 [18]	January 2015–May 2019	26	local	94				49			
Etard, C. et al. (2017) [37]	2016	427	nacional	130	190	1718	2770	37.2	58		
Isoardi, P et al. (2019) [38]	January 2015–January 2019	489		164.5		2197		32.1			
Chun. CW et al. (2014) [39]		111	local	-	272.8				61.1		276
Rizk. C at al. (2021) [20]	March 2016–December 2019	39	local	91		1113		15		288	
Choi, J et al. (2019) [40]	January 2012–June 2014		local		206.2		4240		60		334

### Limitations

The single-centre, retrospective dose audit and DRL analysis study design inherently limits external validity and causal inference. All procedures were performed by one highly experienced neurointerventionalist with long-standing familiarity with low-frame-rate imaging; therefore, reproducibility in less specialized settings and among less experienced operators remains uncertain. No contemporaneous control group using standard frame-rate protocols was available, which precludes direct causal attribution of the observed low dose levels to the low-dose strategy or frame-rate reduction alone. This was because the routine clinical workflow on this system used the low-dose configuration throughout the study period, and conventional frame rates were not applied in practice. A prospective protocol-comparison study requiring dose escalation in a subgroup solely for research purposes was not undertaken, as it would expose patients to additional radiation without direct clinical benefit. In addition, some subgroup comparisons involved small sample sizes (e.g., posterior circulation subgroups) and may be underpowered; these analyses should therefore be interpreted as exploratory.

Finally, all procedures were performed on a single biplane angiography system using vendor-specific dose-reduction and dose-optimization features; thus, generalizability across different platforms and configurations is limited. Multicenter, multi-operator validation is required before extrapolating these DRLs as wider reference standards.

We analyzed radiation exposure only during the endovascular procedure. Follow-up CT imaging performed during hospitalization was not included and should be considered when evaluating cumulative patient exposure.

No formal, blinded, multi-reader image-quality assessment (e.g., contrast-to-noise ratio [CNR], standardized criteria) or clinical endpoint analysis was performed. The retrospective SNR calculation in 15 consecutive low-dose cases (mean 52.7, SD 23.0) is presented as supportive information only and does not constitute a comprehensive validation of diagnostic performance or procedural safety.

Detailed aneurysm morphology, procedural complexity metrics, and cranial anthropometric measures were not available in a structured manner across the retrospective dataset, limiting multivariable adjustment and quantification of potential confounding. We also did not quantify a learning curve or time-trend effect (dose over time) within the study period; such analyses would require a dedicated design with case-mix control.

In our center, coil-only treatment is typically reserved for anatomically favorable aneurysms; therefore, case selection may contribute to lower observed dose levels and should be considered when interpreting comparisons with more heterogeneous endovascular series.

## 5. Conclusions

In this single-centre cohort of coil-only intracranial aneurysm embolization procedures performed between 2018 and 2024 on a modern biplane angiography system using a dedicated low-dose protocol, we established local DRLs for DAP and Ka, r within the lower range of published dose metrics; however, literature comparisons are descriptive and should be interpreted cautiously due to methodological heterogeneity across studies.

The low dose levels observed likely reflect a combination of very low fluoroscopy and DSA frame rates, advanced dose-reduction technology, and extensive operator experience with low-dose imaging. Our findings support the feasibility of achieving low procedural dose levels and defining local DRLs in appropriately selected coil-only cases within a consistent workflow; however, this retrospective audit was not designed to validate clinical safety endpoints, quantify a learning curve, or isolate the independent effect of frame-rate reduction from other factors (equipment configuration, operator practice, and case selection). Further multicenter, multi-operator studies are required to validate these observations and support broader implementation of ambitious local DRLs.

## Figures and Tables

**Table 1:** Number of aneurysm treatment procedures by sex, age, and height.

Variable	Female	Male	Total
Mean age [years] ± SD *	60 ± 12	56 ± 13	59 ± 13
Mean height [m **] ± SD	1.57 ± 0.28	1.72 ± 0.21	1.62 ± 0.27
Mean body weight [kg ***] ± SD	67.59 ± 19.04	80.87 ± 17.74	71.66 ± 19.6
Number of procedures	170	75	245

SD *—standard deviation, m **—meter, kg ***—kilogram.

**Table 2:** Results for all 245 aneurysmal treatments, subdivided into: female, male, RIA, UIA, frontal lamp only, anterior location, posterior location frontal and lateral lamp.

Variable	Nr	DAP			*p*	K_ar_			*p*	Number of Images			*p*	FT Fluoroscopy Time in Seconds			*p*
		P50	P75	Mean ± SD		P50	P75	Mean ± SD		P50	P75	Mean ± SD		P50	P75	Mean ± SD	
all	245	13.8	22.4	19.6 ± 15.5		196.0	268.0	226.0 ± 135.0		208.0	285.0	243.7 ± 174.0		805.0	1138.0	935.6 ± 478.1	
Female	170	13.2	20.2	18.1 ± 14.1	*p* < 0.05	190	260.7	209.2 ± 119.7	*p* < 0.05	208.5	284.5	244.0 ± 176.2	0.89	801.5	1105.5	950.0 ± 504.2	0.59
Male	75	16.2	26.8	22.9 ± 17.9		232	317	264.0 ± 158.9		198	289	242.8 ± 168.6		815	1139	902.9 ± 414.2	
SAH/RIA	115	14.9	22.7	20.32 ± 14.48	*p* < 0.05	218	326.5	258.1 ± 143.1	*p* < 0.05	168	264.5	246.8 ± 217.9	*p* < 0.05	712	992.5	818.5 ± 403.4	*p* < 0.05
UIA	130	13.4	22.3	18.90 ± 16.45		168.5	259.5	197.6 ± 121.0		229	301.5	240 ± 122.6		908	1336	908.5 ± 515.2	
Frontal lamp only	95	12.0	16.9	16.7 ± 14.5	*p* < 0.05	122.0	172.5	134.5 ± 53.9	*p* < 0.05	244.0	309.5	255.5 ± 139.7	*p* < 0.05	891.0	1165.5	954.6 ± 414.0	0.23
Frontal and lateral lamp	150	15.8	24.0	21.4 ± 37.3		245.5	341.5	284.0 ± 422.8		169.0	253.0	2336.2 ± 428.3		791.0	1064.0	923.5 ± 1439.1	
Anterior	180	13.6	20.2	18.7 ± 15.3	0.14	197	269.25	221.8 ± 129.0	0.44	198.5	257	225.7 ± 162.3	*p* < 0.05	810	1100.5	952.0 ± 500.0	0.40
posterior	65	16.2	24.9	21.7 ± 16.0		197	257	238.6 ± 151.5		246	309	290.0 ± 195		799	1052	885.0 ± 408.3	

## Data Availability

No new data were created.

## Supplementary Materials

The following supporting information can be downloaded at https://www.mdpi.com/article/10.3390/biomedicines14010233/s1, Table S1. Number of aneurysm treatment procedures by patient characteristics (age, sex), aneurysm location. Table S2. Percentage distribution of 3D-rotational angiography (3D-RA) utilization in procedures, stratified by number of X-ray tubes/projections (single vs dual) and aneurysm status (UIA vs RIA). Table S3. Results for all 245 aneurysmal treatments, subdivided into: female, male, RIA female, UIA female, RIA male and UIA male frontal lamp only and frontal and lateral lamp. Table S4. Probability results for differences between aneurysmal treatment subgroups (female RIA, Male RIA, female UIA, Male UIA, frontal lamp only or frontal and lateral lamp) for dose area product (DAP), air kerma (K_a,r_), fluoroscopy time (FT) and total digital subtraction angiography (DSA) frames. Table S5. Results for aneurysmal treatments, subdivided into anterior and posterior circulation of the circle of Willis and UIA and RIA in these subgroups. Table S6. Probability results for differences between aneurysmal treatment subgroups (anterior vs posterior circulation of the circle of Willis, and subgroups: UIA, RIA) for dose area product (DAP), air kerma (K_a,r_), fluoroscopy time (FT) and total digital subtraction angiography (DSA) frames.
