# Supplementary material for: Local Diagnostic Reference Levels for Intracranial Aneurysm Coil-Only Embolization Using a Low-Dose Technique
Source: Biomedicines. 2026 Jan 21;14(1):233. doi: 10.3390/biomedicines14010233 (PMC12838866; doi:10.3390/biomedicines14010233)
Supplement: Supplementary file 1 [file biomedicines-14-00233-s001.zip › biomedicines-4082160-supplementary.pdf]

Supplement the manuscript ID biomedicines-4021719 Local diagnostic reference levels for intracranial aneurysm coil-only embolization using a low-dose te
chnique.

Table S1. Number of aneurysm treatment procedures by patient characteristics (age, sex), aneurysm location.

		UIA*	RIA**	Total
Patient characteristic	Patient numer	130	115	245
	Sex	101 females<br>29 males	69 females<br>46 males	170 females<br>75 males
	Age (mean) range ± SD	60 ±11	57 ±12	59±13
Aneurysm location	Anterior cilculation	103	77	180
	Posterior circulation	28	37	65
Multiple aneurysms		28	15	49
2 aneurysms treated during a single endovascular session		6	2	8
3 aneurysms treated during a single endovascular session		1	1	2

UIA- unruptured intracranial aneurysms
RIA- ruptured intracranial aneurysms
Three-Dimensional Rotational Angiography

Table S2. Percentage distribution of 3D-rotational angiography (3D-RA) utilization in procedures, stratified by number of X-ray tubes/projections (single vs dual) and aneurysm status (UIA vs RIA).

X-ray tubes/projections	Frontal only		Frontal and lateral		all
	RIA	UIA	RIA	UIA	
Number 3D-RA per procedure	9/22	64/73	2/93	4/57	79/245
% all (245)	3.7%	26.1%	0.8 %	1.6%	32.2%
% all with 3D-RA(79)	11.4%	81.0%	2.5%	5.1%	100%

Table S3. Results for all 245 aneurysmal treatments, subdivided into: female, male, RIA female, UIA female, RIA male and UIA male frontal lamp only and frontal and lateral lamp.

Variable	Nr	DAP				K_{ar}				Number of images				FT<br>Fluoroscopy time in seconds			
		P25	P50 typical values	P75	Mean ± SD	P25	P50 typical values	P75	Mean ± SD	P25	P50 typical values	P75	Mean ± SD	P25	P50 typical values	P75	Mean ± SD
all	245	9.7	13.8	22.4	19.6 ±15.5	127.0	196.0	268.0	226.0 ±135.0	140.0	208.0	285.0	243.7 ±174.0	623.0	805.0	1138.0	935.6 ±478.1
Female	170	9.5	13.2	20.2	18.1 ±14.1	121.5	190	260.7	209.2 ±119.7	143.2	208.5	284.5	244.0 ±176.2	633.5	801.5	1105.5	950.0 ±504.2
Male	75	12.3	16.2	26.8	22.9 ±17.9	161	232	317	264.0 ±158.9	128	198	289	242.8 ± 168.6	583	815	1139	902.9 ±414.2
Female SAH/ RIA	69	9	13	20	18.9 ±13.3	161	205	255	237.9 ±124.0	118	166	246	255 ±240	585	712	865	814 ±436
Male SAH/ RIA	46	12.7	16.1	22.1	22.5 ±16.0	171.2	249.5	323.2	188 ±164.4	107.5	169.5	257.75	233.2 ±181	561.75	709.5	1001.5	824.8 ±353.0
Male UIA	29	12.0	17.9	26.5	20. 7 ±21	124	191	243	225.4 ±144	153	250	318	258.1 ±144	677	909	1145	1027 ±477
Female UIA	101	8.8	12.9	17.4	17.5 ±14.7	96.9	164	258	189.5 ±113.0	164	225	285	236.0 ±114.5	699	908	1290	1043.0 ±528.0
Frontal lamp only	95	8.6	12.0	16.9	16.7 ±14.5	93.9	122.0	172.5	134.5 ±53.9	199.0	244.0	309.5	255.5 ±139.7	643.5	891.0	1165.5	954.6 ±414.0
Frontal and lateral lamp	150	11.4	15.8	24.0	21.4 ±37.3	190.3	245.5	341.5	284.0 ±422.8	124.3	169.0	253.0	2336.2 ±428.3	610.0	791.0	1064.0	923.5 ±1439.1
Frontal lamp only SAH/ RIA	22	8.9	12.4	14.5	15.3 ±11.2	106.0	135.0	183.0	149.3 ±47.2	85.8	179.0	283.0	239.2 ± 219.5	529.5	629.0	827.8	743.5 ±353.6
Frontal and lateral lamp SAH/ RIA	93	11.4	16.3	24.9	21.5 ±15.0	189.0	238.0	350.0	283.9 ±146.1	118.0	161.0	260.0	248.6 ±218.6	593.0	714.0	1002.0	836.2 ±414.1

Frontal lamp only UIA	73	8.3	11.5	17.2	17.2 ±15.4	91.4	112.5	166.3	130.0 ±55.3	208.0	248.5	313.5	260.4 ±106.4	699.0	922.0	1315.0	1018.9 ± 411.8
Frontal and lateral lamp UIA	57	11.4	14.6	23.1	21.1 ±17.6	197.0	262.0	335.0	284.1 ± 1274	136.0	185.5	253.0	216 ±137.8	677.0	870.0	1348.0	1066.0 ±626.2

Table S4. Probability results for differences between aneurysmal treatment subgroups (female RIA, Male RIA, female UIA, Male UIA, frontal lamp only or frontal and lateral lamp) for dose area product (DAP), air kerma ($K_{a,r}$), fluoroscopy time (FT) and total digital subtraction angiography (DSA) frames.

Variable	Test	p-value	The test used
DAP	Female vs male	P<0.05	Mann-Whitney
	female RIA vs male RIA	0.11	Mann-Whitney
	female RIA vs female UIA	0.24	Mann-Whitney
	male RIA vs male UIA	0.98	Mann-Whitney
	male UIA vs female UIA	P<0.05	Mann-Whitney
	Frontal lamp only vs Frontal and lateral lamp	P<0.05	Mann-Whitney
K	Female vs male	P<0.05	T-Student
	female RIA vs male RIA	0.09	T-Student
	female RIA vs female UIA	P<0.05	T-Student
	male RIA vs male UIA	P<0.05	T-Student
	male UIA vs female UIA	0.14	T-Student
	Frontal lamp only vs Frontal and lateral lamp	P<0.05	T-Student
FT	Female vs male	0.59	Mann-Whitney
	female RIA vs male RIA	0.68	T-Student
	female RIA vs female UIA	P<0.05	T-Student
	male RIA vs male UIA	P<0.05	T-Student
	male UIA vs female UIA	0.85	T-Student
	Frontal lamp only vs Frontal and lateral lamp	0.23	Mann-Whitney
Number of images	Female vs male	0.89	Mann-Whitney
	female RIA vs male RIA	0.92	Mann-Whitney
	female RIA vs female UIA	P<0.05	Mann-Whitney
	male RIA vs male UIA	0.20	T-Student
	male UIA vs female UIA	0.50	Mann-Whitney
	Frontal lamp only vs Frontal and lateral lamp	P<0.05	Mann-Whitney

Table S5. Results for aneurysmal treatments, subdivided into anterior and posterior circulation of the circle of Willis and UIA and RIA in these subgroups.

Aneurysm location	Nr	DAP				K_{ar}				Number of images				FT Fluoroscopy time in seconds			
		P25	P50 typical values	P75	Mean ± SD	P25	P50 typical values	P75	Mean ± SD	P25	P50 typical values	P75	Mean ± SD	P25	P50 typical values	P75	Mean ± SD
RIA	115	10.4	14.9	22.7	20.3 ±14.5	168.5	218	326.5	258.1 ±143.1	110	168	264.5	246.8 ±217.9	572	712	992.5	818.5 ±403.4
UIA	130	9.5	13.4	22.3	18.9 ±16.5	103.5	168.5	259.5	197.6 ±121.0	163.3	229	301.5	240.9 ±122.7	699	908.5	1336	1039.2 ±515.2
Anterior	180	9.7	13.6	20.2	18.7 ±15.3	124	197	269.25	221.8 ±129.0	130.25	198.5	257	225.7 ±162.3	628.5	810	1100.5	952.0 ±500.0
posterior	65	9.7	16.2	24.9	21.7 ±16.0	140	197	257	238.6 ±151.5	154	246	309	290.0 ±195	598	799	1052	885.0 ±408.3
Anterior RIA	77	10.3	13.8	20.5	19.0 ±12.9	164.25	224	302.75	251.9 ±135.8	102.25	154.5	243	217.5 ±196.3	586.5	712.5	983.5	851.4 ±447.1
Anterior UIA	83	9.5	13.3	18.4	18.5 ±17.0	106	178.5	260.75	199.0 ±118.5	150.75	215.5	265.25	232.0 ±131.2	678.75	908.5	1270.5	1028.7 ±526.2
Posterior RIA	37	10.6	16.2	43.6	23.0 ±17.3	174	218	343	271.7 ±158.3	146	194	628	308.4 ±249.2	539	681	973.5	749 ±283.6
Posterior UIA	28	9.1	16.0	25.0	20.2 ±14.5	100.5	158.5	241	192.7 ±131.7	232.7	291.5	337	273.1 ±78.0	701.2	953.5	1433.5	1077.3 ±480.1

Table S6. Probability results for differences between aneurysmal treatment subgroups (anterior vs posterior circulation of the circle of Willis, and subgroups: UIA, RIA) for dose area product (DAP), air kerma ($K_{a,r}$), fluoroscopy time (FT) and total digital subtraction angiography (DSA) frames.

	Nr of patients	variable	p-value	the test used
RIA vs UIA	115 vs 130	DAP	0.11	Mann-Whitney
		K	$p < 0.05$	T-Student
		FT	$p < 0.05$	T-Student
		Number of images	$p < 0.05$	Mann-Whitney
anterior vs posterior circulation of the circle of Willis	180 vs 65	DAP	0.14	Mann-Whitney
		K	0.44	T-Student
		FT	0.40	Mann-Whitney
		Number of images	$p < 0.05$	Mann-Whitney
anterior circulation of the circle of Willis : RIA vs UIA	RIA 77 vs UIA 103	DAP	0.15	Mann-Whitney
		K	$p < 0.05$	T-Student
		FT	$p < 0.05$	T-Student
		Number of images	$p < 0.05$	Mann-Whitney
posterior circulation of the circle of Willis : RIA vs UIA	37 RIA vs 28 UIA	DAP	0.71	Mann-Whitney
		K	$p < 0.05$	T-Student
		FT	$p < 0.05$	T-Student
		Number of images	$p < 0.05$	Mann-Whitney
RIA posterior vs RIA anterior circulation of the circle of Willis:	RIA posterior 37 RIA anterior 77	DAP	0.44	Mann-Whitney
		K	0.50	T-Student
		FT	0.24	T-Student
		Number of images	$p < 0.05$	Mann-Whitney
UIA posterior vs UIA anterior circulation of the circle of Willis:	UIA anterior 103 UIA posterior 28	DAP	0.23	Mann-Whitney
		K	0.75	T-Student
		FT	$p < 0.05$	T-Student
		Number of images	$p < 0.05$	Mann-Whitney
